# Supplementary material for: A New 3-D Open-Framework Zinc Borovanadate with Catalytic Potentials in α-Phenethyl Alcohol Oxidation
Source: Molecules. 2019 Feb 1;24(3):531. doi: 10.3390/molecules24030531 (PMC6384951; doi:10.3390/molecules24030531)
Supplement: Supplementary file 1 [file molecules-24-00531-s001.pdf]

Article

A New 3-D Open-Framework Zinc Borovanadate with Catalytic Potentials in α-Phenethyl Alcohol Oxidation

Xinxin Liu [1,2], Biao Guo [2], Xuejiao Sun [1], Le Zhang [1] and Hongming Yuan [1,*]

1. State Key Laboratory of Inorganic Synthesis and Preparative Chemistry, College of Chemistry, Jilin University, Changchun 130012, China; liuxinxin1114@163.com (X.L.); sunxj17@jlu.edu.cn (X.S.); zhangle16@jlu.edu.cn (L.Z)
2. Institute of Catalysis for Energy and Environment, College of Chemistry and Chemical Engineering, Shenyang Normal University, Shenyang 110034, China; biaoguo14@126.com

* Correspondence: hmyuan@jlu.edu.cn (H.Y.); Tel: +86-0431-8516-8318 (H.Y.)

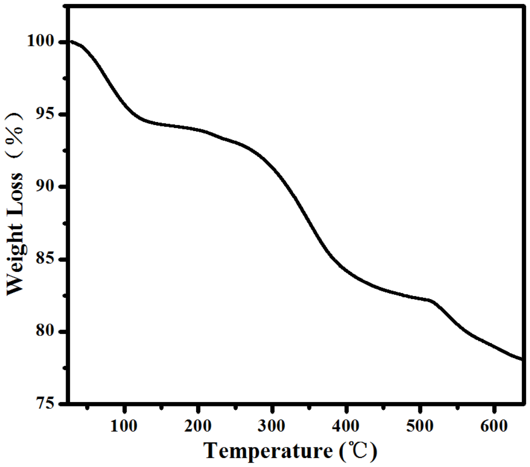

Figure S1. TG curve of **1** in N_2.

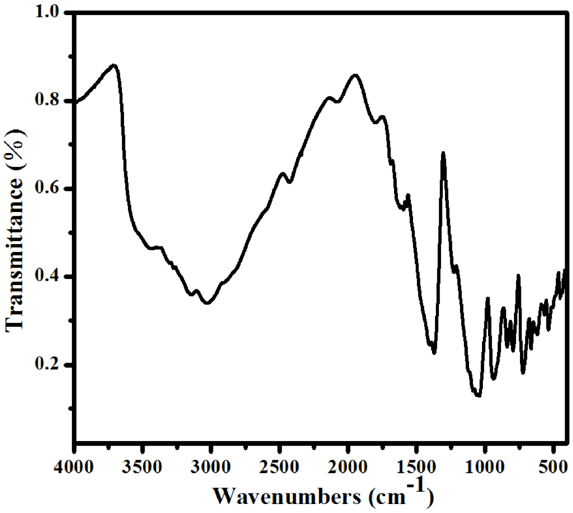

Figure S2. The FT-IR spectrum of **1**.

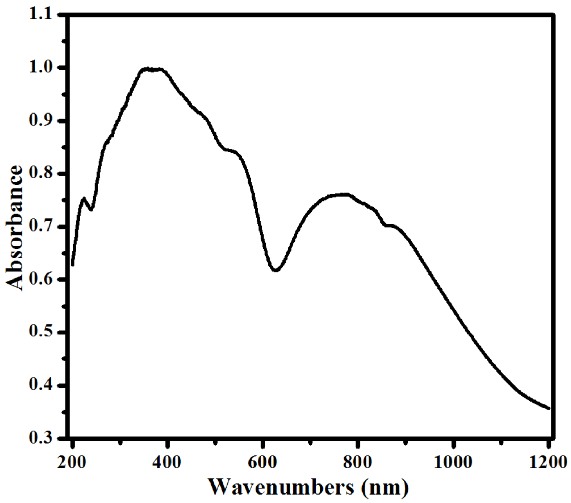

Figure S3. UV-Vis-NIR diffuse reflectance spectrum of **1**.

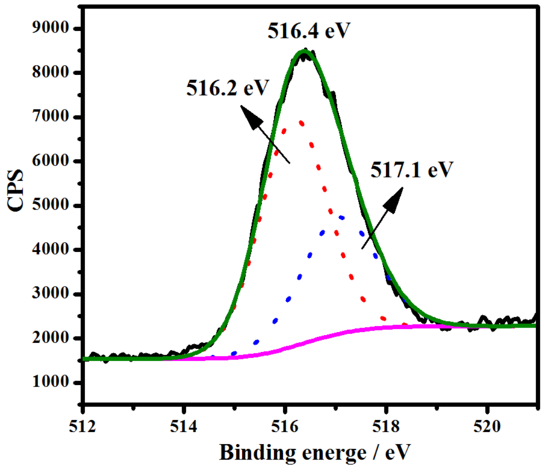

Figure S4. XPS spectrum of vanadium in **1**. The peak at 516.2 eV is attributed to V^{4+} and a shoulder at about 517.1 eV is assigned to for V^{5+}

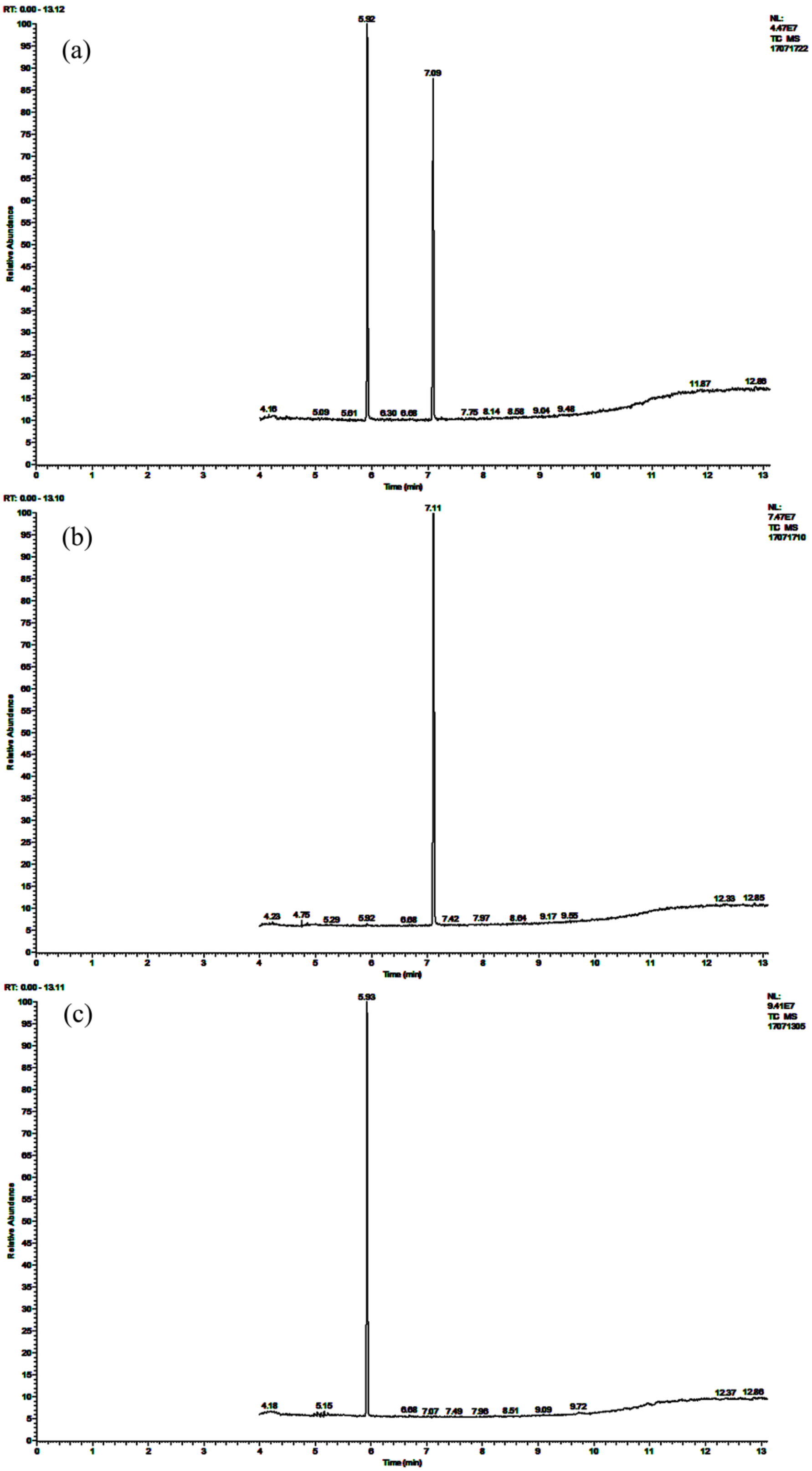

Figure S5. The chromatogram for the oxidation of α-phenethyl alcohol to acetophenone. (a) products; (b) α-phenethyl alcohol standard sample;(c) acetophenone standard sample

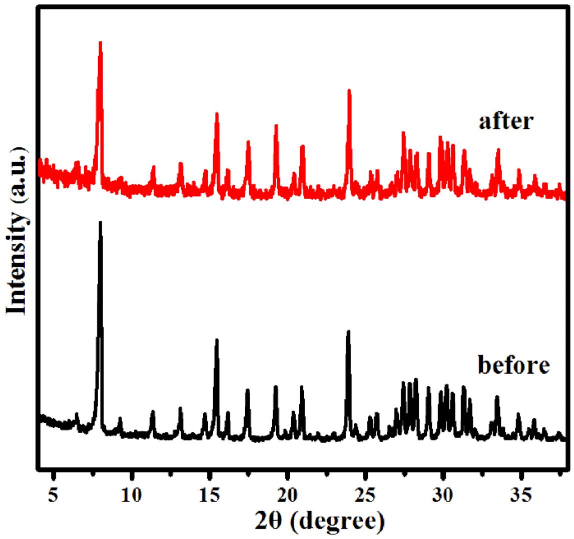

Figure S6. The PXRD patterns of compound **1** collected beforofe and after the catalytic reactions.

Table S1. Comparison of cyrstal structure of compound **1** obtained in this study with other reprehensive reports on three-dimensional open-framework borovanadates

Compound	borovanadate cluster	*bridges*	Ref.
SUT-6-Zn	$(VO)_{12}O_6B_{18}O_{36}(OH)_6$	ZnO_5	[17]
SUT-6-Mn	$(VO)_{12}O_6B_{18}O_{36}(OH)_6$	MnO_6	[17]
SUT-6-Ni	$(VO)_{12}O_6B_{18}O_{36}(OH)_6$	NiO_6	[17]
SUT-7-Zn	$(V_{10}B_{28}O_{74}H_8)^{8-}$	ZnO_5	[7]
$\{[Cu(dien)(H_2O)]_3V_{12}B_{18}O_{54}(OH)_6(H_2O)\}\cdot 4H_3O\cdot 5.5H_2O$	$(VO)_{12}O_6B_{18}O_{36}(OH)_6$	$Cu(en)_2$	[32]
$[Cu^{II}(en)_2]_4\{Na(H_2O)(\mu\text{-}OH)[B(OH)_2]\}_2[(V^{V}O)_2(V^{IV}O)_{10}O_6(B_{18}O_{36}(OH)_6)]\}\cdot 7H_2O$	$(VO)_{12}O_6B_{18}O_{36}(OH)_6$	$Cu(en)_2$, $Na(H_2O)(\mu\text{-}OH)[B(OH)_2]$	[36]
$[Cd_3(H_2O)_6][(VO)_6(VO)_6O_6)(B_{18}O_{36}(OH)_6)]\cdot 10H_2O$	$(VO)_{12}O_6B_{18}O_{36}(OH)_6$	$Cd(H_2O)_2O_4$	[45]
$[Zn_6(en)_3][(V^{IV}O)_6(V^{V}O)_6O_6(B_{18}O_{36}(OH)_6)\cdot(H_2O)]_2\cdot 14H_2O$	$(VO)_{12}O_6B_{18}O_{36}(OH)_6$	$Zn(en)$	In this work

Table S2. Bond lengths [Å] for **1**.

Zn(1)-O(1)	1.995(3)
Zn(1)-O(1)#1	1.995(3)
Zn(1)-N(1)#1	2.035(9)
Zn(1)-N(1)	2.035(9)
Zn(1)-O(10)#1	2.199(3)
Zn(1)-O(10)	2.199(3)
V(1)-O(3)	1.609(3)
V(1)-O(6)	1.941(3)
V(1)-O(4)	1.953(3)
V(1)-O(5)	1.957(3)
V(1)-O(2)	2.021(3)
V(1)-V(2)	3.0065(10)
V(1)-V(2)#2	3.0127(10)
V(2)-O(7)	1.631(3)
V(2)-O(6)	1.927(3)
V(2)-O(6)#3	1.930(3)
V(2)-O(5)#3	1.953(3)
V(2)-O(4)	1.966(3)
V(2)-V(1)#3	3.0127(10)
O(4)-B(2)	1.448(5)
O(2)-B(2)#4	1.492(5)
O(2)-B(1)	1.501(5)
O(5)-B(1)#4	1.467(5)
O(5)-V(2)#2	1.953(3)
O(8)-B(3)	1.358(5)
O(8)-B(1)#5	1.473(5)
O(6)-V(2)#2	1.930(3)
O(9)-B(3)	1.353(6)
B(3)-O(10)	1.375(5)
B(2)-O(1)	1.449(5)
B(2)-O(2)#5	1.492(5)
B(2)-O(10)	1.507(5)
O(1)-B(1)	1.442(5)
B(1)-O(5)#5	1.467(5)
B(1)-O(8)#4	1.473(5)
N(1)-N(1)#1	0.836(17)
N(1)-C(1)	1.072(14)
N(1)-C(1)#1	1.490(14)
C(1)-N(1)#1	1.490(14)
C(1)-C(1)#6	1.59(2)
C(1)-C(1)#1	1.91(2)

Table S3. Bond angles [deg] for **1**.

O(1)-Zn(1)-O(1)#1	149.98(16)
O(1)-Zn(1)-N(1)#1	104.4(3)
O(1)#1-Zn(1)-N(1)#1	105.0(3)
O(1)-Zn(1)-N(1)	105.0(3)
O(1)#1-Zn(1)-N(1)	104.4(3)
N(1)#1-Zn(1)-N(1)	23.7(5)
O(1)-Zn(1)-O(10)#1	102.47(11)
O(1)#1-Zn(1)-O(10)#1	65.46(10)
N(1)#1-Zn(1)-O(10)#1	123.7(3)
N(1)-Zn(1)-O(10)#1	101.4(3)
O(1)-Zn(1)-O(10)	65.46(10)
O(1)#1-Zn(1)-O(10)	102.47(11)
N(1)#1-Zn(1)-O(10)	101.4(3)
N(1)-Zn(1)-O(10)	123.7(3)
O(10)#1-Zn(1)-O(10)	134.75(16)
O(3)-V(1)-O(6)	109.22(16)
O(3)-V(1)-O(4)	108.32(15)
O(6)-V(1)-O(4)	78.71(12)
O(3)-V(1)-O(5)	110.00(15)
O(6)-V(1)-O(5)	78.09(12)
O(4)-V(1)-O(5)	139.92(12)
O(3)-V(1)-O(2)	107.27(16)
O(6)-V(1)-O(2)	143.51(12)
O(4)-V(1)-O(2)	90.30(11)
O(5)-V(1)-O(2)	89.33(11)
O(3)-V(1)-V(2)	111.72(13)
O(6)-V(1)-V(2)	38.81(8)
O(4)-V(1)-V(2)	40.07(8)
O(5)-V(1)-V(2)	112.28(9)
O(2)-V(1)-V(2)	123.94(8)
O(3)-V(1)-V(2)#2	112.39(13)
O(6)-V(1)-V(2)#2	38.77(8)
O(4)-V(1)-V(2)#2	113.00(9)
O(5)-V(1)-V(2)#2	39.54(8)
O(2)-V(1)-V(2)#2	123.04(8)
V(2)-V(1)-V(2)#2	75.72(3)
O(7)-V(2)-O(6)	109.04(15)
O(7)-V(2)-O(6)#3	107.59(15)
O(6)-V(2)-O(6)#3	92.71(17)
O(7)-V(2)-O(5)#3	107.78(15)
O(6)-V(2)-O(5)#3	143.10(13)

O(6)#3-V(2)-O(5)#3	78.45(12)
O(7)-V(2)-O(4)	108.53(15)
O(6)-V(2)-O(4)	78.73(12)
O(6)#3-V(2)-O(4)	143.72(12)
O(5)#3-V(2)-O(4)	87.50(12)
O(7)-V(2)-V(1)	111.74(12)
O(6)-V(2)-V(1)	39.15(9)
O(6)#3-V(2)-V(1)	125.26(9)
O(5)#3-V(2)-V(1)	121.35(9)
O(4)-V(2)-V(1)	39.75(8)
O(7)-V(2)-V(1)#3	109.86(12)
O(6)-V(2)-V(1)#3	125.16(9)
O(6)#3-V(2)-V(1)#3	39.03(9)
O(5)#3-V(2)-V(1)#3	39.64(8)
O(4)-V(2)-V(1)#3	121.74(9)
V(1)-V(2)-V(1)#3	138.34(3)
B(2)-O(4)-V(1)	130.0(2)
B(2)-O(4)-V(2)	129.7(2)
V(1)-O(4)-V(2)	100.18(13)
B(2)#4-O(2)-B(1)	112.7(3)
B(2)#4-O(2)-V(1)	122.1(2)
B(1)-O(2)-V(1)	123.0(2)
B(1)#4-O(5)-V(2)#2	127.3(2)
B(1)#4-O(5)-V(1)	131.9(2)
V(2)#2-O(5)-V(1)	100.82(13)
B(3)-O(8)-B(1)#5	124.5(3)
V(2)-O(6)-V(2)#2	146.58(17)
V(2)-O(6)-V(1)	102.03(13)
V(2)#2-O(6)-V(1)	102.20(13)
O(9)-B(3)-O(8)	117.3(4)
O(9)-B(3)-O(10)	122.3(4)
O(8)-B(3)-O(10)	120.4(4)
O(4)-B(2)-O(1)	112.0(3)
O(4)-B(2)-O(2)#5	109.7(3)
O(1)-B(2)-O(2)#5	113.3(3)
O(4)-B(2)-O(10)	112.1(3)
O(1)-B(2)-O(10)	100.6(3)
O(2)#5-B(2)-O(10)	108.8(3)
B(1)-O(1)-B(2)	119.4(3)
B(1)-O(1)-Zn(1)	136.6(2)
B(2)-O(1)-Zn(1)	102.0(2)
B(3)-O(10)-B(2)	119.4(3)
B(3)-O(10)-Zn(1)	140.8(3)
B(2)-O(10)-Zn(1)	91.5(2)

O(1)-B(1)-O(5)#5	109.0(3)
O(1)-B(1)-O(8)#4	107.2(3)
O(5)#5-B(1)-O(8)#4	110.8(3)
O(1)-B(1)-O(2)	112.5(3)
O(5)#5-B(1)-O(2)	109.3(3)
O(8)#4-B(1)-O(2)	107.9(3)
N(1)#1-N(1)-C(1)	102.0(13)
N(1)#1-N(1)-C(1)#1	44.7(9)
C(1)-N(1)-C(1)#1	94.8(12)
N(1)#1-N(1)-Zn(1)	78.1(2)
C(1)-N(1)-Zn(1)	138.6(10)
C(1)#1-N(1)-Zn(1)	111.3(6)
N(1)-C(1)-N(1)#1	33.3(8)
N(1)-C(1)-C(1)#6	136.9(8)
N(1)#1-C(1)-C(1)#6	112.5(8)
N(1)-C(1)-C(1)#1	51.1(8)
N(1)#1-C(1)-C(1)#1	34.1(6)
C(1)#6-C(1)-C(1)#1	86.1(4)

Symmetry transformations used to generate equivalent atoms:

#1 -x+3/2,-y+1/2,z #2 -z+1/2,x,-y+1/2

#3 y,-z+1/2,-x+1/2 #4 -y+1,z+1/2,x-1/2

#5 z+1/2,-x+1,y-1/2 #6 -x+3/2,y,-z+1/2
